# Comparison of Wear of Interim Crowns in Accordance with the Build Angle of Digital Light Processing 3D Printing: A Preliminary In Vivo Study

**DOI:** 10.3390/bioengineering9090417

**Published:** 2022-08-25

**Authors:** Hakjun Lee, Keunbada Son, Du-Hyeong Lee, So-Yeun Kim, Kyu-Bok Lee

**Affiliations:** 1 Department of Prosthodontics, School of Dentistry, Kyungpook National University, Daegu 41940, Korea; 2 Advanced Dental Device Development Institute (A3DI), Kyungpook National University, Daegu 41940, Korea

**Keywords:** 3D printing, digital light processing, interim crown, in vivo study, wear

## Abstract

The aim of this study is to evaluate the wear volume of interim crowns fabricated using digital light processing 3D printing according to the printing angle. A total of five patients undergoing the placement of a single crown on the mandibular molar were included. Interim crowns were fabricated directly in the oral cavity using the conventional method. A digital light processing 3D printer was then used to fabricate crowns with build angles of 0, 45, and 90 degrees. Therefore, four fabricated interim crowns were randomly delivered to the patients, and each was used for one week. Before and after use, the intaglio surfaces of the interim crowns were scanned using a 3D scanner. The volume changes before and after use were measured, and changes in the height of the occlusal surface were evaluated using the root mean square value. Data normality was verified by statistical analysis, and the wear volume in each group was evaluated using a one-way analysis of variance and Tukey’s honestly significant difference test (α = 0.05). Compared with the RMS values of the conventional method (11.88 ± 2.69 µm) and the 3D-printing method at 0 degrees (12.14 ± 2.38 µm), the RMS values were significantly high at 90 degrees (16.46 ± 2.39 µm) (*p* < 0.05). Likewise, there was a significant difference in the change in volume between the groups (*p* = 0.002), with a significantly higher volume change value at 90 degrees (1.74 ± 0.41 mm^3^) than in the conventional method (0.70 ± 0.15 mm^3^) (*p* < 0.05). A printing angle of 90 degrees is not recommended when interim crowns are fabricated using digital light processing 3D printing.

## 1. Introduction

Interim crowns are important for successful prosthetic restoration. Interim crowns provide essential functional support, including proper maintenance of the occlusal relationship, prevention of tooth movement, and protection of the dental pulp and periodontal tissue [1,2]. The appropriate wear resistance of interim crowns is required to maintain these functions. If wear is excessive, there may be a change in the functionality of the masticatory movement as the occlusal vertical dimension decreases. In addition, masticatory efficiency may decrease, and premature contact may occur in the anterior teeth [3].

The conventional direct technique for an interim crown is a method of manufacturing using a material in which resin polymerization occurs only by chemical catalysis without heat or light, and 3D printing with photopolymerization technology is a method of activating a photo initiator through a light source [4]. In the conventional method, interim crowns are directly manufactured in the oral cavity [5]. However, such interim crowns may be affected by high rates of contraction and heat production and lack mechanical characteristics, unlike crowns fabricated using the indirect method with computer-aided design and computer-aided manufacturing (CAD/CAM) technologies, known as the milling production method [6,7,8]. Crowns fabricated using the milling production method have superior wear and flexure strength [9], short chair time placement, and superior marginal and internal fit compared with those of crowns fabricated using the conventional method [8]. Recently, studies on the production of interim crowns using 3D printing have been conducted. Among these methods, a stereolithography apparatus (SLA) and digital light processing (DLP) are representative methods [10]. The main difference between an SLA and DLP is the light source. An SLA applies a laser beam from point to point, while DLP uses a digital micromirror to cure a complete resin layer-by-layer [11]. Unkovskiy et al. [12] reported for accuracy that an SLA may produce an intaglio denture surface with a better trueness than DLP. Li et al. [11] reported that the surface roughness was significantly influenced by the build angle rather than by the AM method. However, DLP has a faster printing speed because the entire layer of liquid resin is polymerized at once, and the DLP method is more actively utilized in the dental field [13].

Various in vitro studies have been conducted on the wear of DLP-printed interim crowns. Kessler et al. [14] investigated the three-body wear of different additively manufactured temporary materials and concluded they had comparative wear resistance to already-established materials. Myagmar et al. [15] measured the wear of DLP-printed and CAD/CAM-milled interim resin materials using a mastication simulator and found these 3D-printing digital technologies exhibited less wear volume loss than the conventional interim resin. These studies on the wear resistance of interim crowns have been conducted in vitro using a mastication simulator because controlling various variables can be difficult [16]. Prause et al. [17] reviewed the wear resistance of 3D-printed materials and could not find any in vivo studies.

A mastication simulator can reflect a variety of elements, such as force, sliding distance, testing medium, pH cycling, and temperature cycling, to imitate the masticatory system of the oral cavity. However, wear is not a simple material property but a system property arising from complex interactions between various factors. The capacity of an in vitro study to take all these factors into account is limited [18]. Indeed, Sari et al. [19] compared in vitro and in vivo wear data of temporary crowns fabricated by a CAD/CAM milling system and a cartridge system. Although the maximum wear differences between in vitro and in vivo were not significant, the mean wear differences between in vitro and in vivo were significant for CAD material. Thus, the clinical wear resistances of 3D-printed interim crowns should be verified in the oral cavity in addition to a mastication simulator.

With the DLP method, the mechanical properties of restoration are affected by the build angle [20]. The difference in physical properties according to the build angle is called anisotropy. Various physical properties of anisotropy have been reported, such as marginal and internal fit [8,21], surface roughness [22], and flexural strength [23]; thus, an optimum printing angle according to each characteristic is recommended. However, few studies on wear according to build angle have been conducted. Hanon et al. [24] studied the effect of build angle on the wear resistance of 3D-printed polymers using a DLP method and concluded no relationship between wear behavior and build angle in a cylinder-shaped specimen. However, in an *X*-axis experiment, the output direction of the layer and the actual sliding direction did not match. Therefore, to date, studies on the relationship between wear and the build angle of interim crowns are still rare.

In summary, there is no in vivo study on the wear resistance of DLP-printed interim crowns. In addition, there are few studies on wear resistance according to the build angle of DLP-printed interim crowns. Therefore, the purpose of this study is to evaluate the wear volume of DLP-printed interim crowns in vivo and to find the build angle with the highest wear resistance among 0, 45, and 90 degrees. In addition, compared with the existing traditional method, the clinical feasibility of DLP-printed interim crowns was demonstrated. The null hypothesis is that the wear volume of DLP-printed interim crowns does not differ according to the build angle.

## 2. Materials and Methods

**Figure 1:**
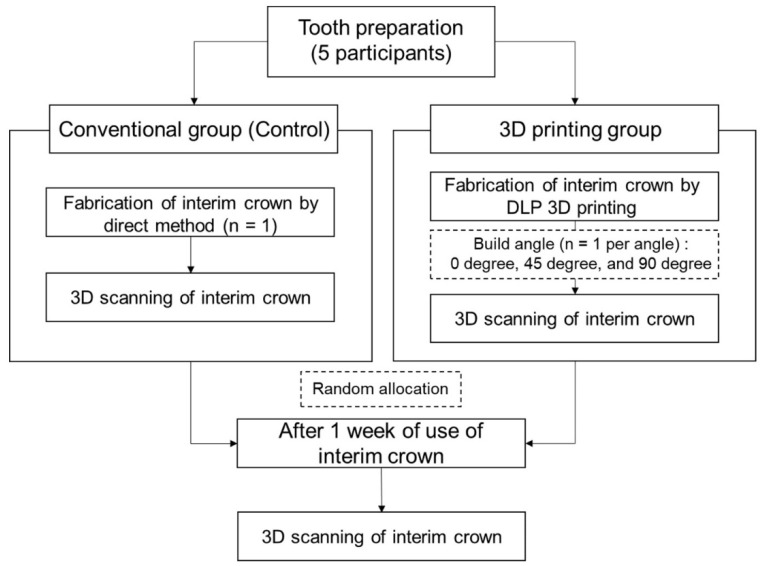
Study design.

This clinical study was approved by the Kyungpook National University Dental Hospital Institutional Review Board (Approval number: KNUDH-2021-11-02-01) (Figure 1). Informed consent was obtained from all participants. Patients who visited the Department of Prosthodontics at Kyungpook National University Dental Hospital for single-crown treatment and consented to participate in the present study were recruited. The selection criteria were as follows: patients who needed single-crown restoration treatment in the mandibular molar teeth, who had no decay or periodontal abnormalities in the abutment tooth, and who had no occlusal problems. Owing to a possible impact on wear or other potential side effects, the following patients were excluded: (1) those requiring restoration of the antagonist teeth, (2) those with a possible cracked tooth, and (3) those with bruxism or clenching habits. Based on the results of a previous study [15], the sample size was calculated to be a minimum of three participants per group (G*Power version 3.1.9.2; Heinrich-Heine-Universität, Düsseldorf, Germany) (actual power = 99.98%; power = 99%; α = 0.05). For the present study, the sample size was determined to be five participants. Anticipating a 20% dropout rate, a total of six patients were recruited. The profiles of the participants are listed in Table 1. To control the variables, each person was asked to use all four types of interim crown so that the same person’s masticatory force was applied to the interim crown for each angle. In addition, patients who need treatment on same area were recruited, and the order of use of interim crown was randomly allocated for each angle. One participant dropped out of the study owing to frequent fractures of the temporary tooth.

At the first visit, mandibular molar teeth were prepared by a skilled technician using a diamond bur (102R bur, Shofu, Kyoto, Japan). During tooth preparation, a supragingival chamfer margin was formed, and 1.5 mm of the occlusal surface was reduced. A skilled operator then obtained a virtual working cast of the abutment tooth, proximal teeth, and antagonist teeth and performed an occlusal scan using an intraoral scanner (CS3600; Carestream Dental, Rochester, NY, USA).

Immediately after tooth preparation, interim crowns were fabricated at the chairside using self-cured resin (Unifast III; GC Corporation, Tokyo, Japan) by the direct conventional method (Table 2). Occlusal adjustment and final polishing were performed by a skilled clinician using the same process for all groups. The interim crowns were polished with silicon carbide papers of 600- and 1200-grit grain on a rotary machine with water cooling. A 21 µm thick check bit (Check-Film II—Red/Black; Caicedo Group Inc., Brooklyn, NY, USA) and an 8 µm thick shimstock (ARI SHIMSTOCK; TAEKWANG, Seoul, Korea) were used to determine whether the occlusal point and height were appropriate. After the final polishing process and immediately before cementation, the interim crown surface was scanned extraorally using an intraoral scanner (CS3600, Carestream Dental). The crowns were temporarily cemented (Temp-bond NE, Kerr, Orange, CA, USA) for one week to avoid sticky or hard foods that might cause falls or fractures of the interim crowns.

**Figure 2:**
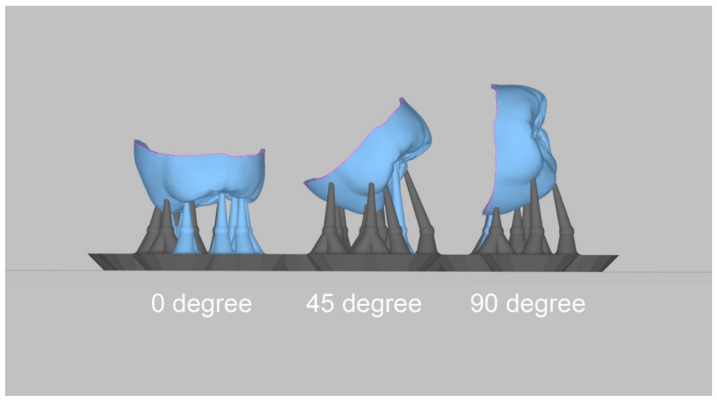
Digital light processing 3D-printing build angles.

The acquired virtual working cast was exported in the standard tesselation language (STL) format using an intraoral scanner. In addition, using CAD software (3Shape Dental Designer; 3Shape A/S, Copenhagen, Denmark), interim crowns were designed in a 60 µm cement space condition. After the CAD process, the virtual interim crowns were exported in the STL format, and the build angles for printing were set to 0, 45, and 90 degrees using 3D printer software (Megagen, Daegu, Korea) (Figure 2). The 3D-printing support was set to the software-recommended value. The crowns were printed using a 3D printer with DLP technology (MEG-PRINTER 3D II; Megagen, Daegu, Korea) under the following conditions: 50 µm XY resolution and 50 µm layer thickness (Table 2). Resin (Raydent C&B; Ray Co., Ltd., Hwaseong-si, Korea) was selected as the 3D-printing material. The printed interim crowns were washed using 83% ethanol, and all residual resin was removed for 60 s using an ultrasonic cleaner (SAEHAN, Seoul, Republic of Korea). All moisture and ethanol on the surface of the interim crown was dried. Finally, after the posttreatment process, a photocuring process was performed for 300 s using a curing unit (CUREDEN; Kwang Myung DAICOM, Seoul, Republic of Korea), and they were stored in distilled water at 37 °C until cementation.

The patients revisited the hospital one week after the first interim crown was placed. The existing interim crown was removed, and the surface was scanned extraorally using an intraoral scanner (CS3600, Carestream Dental, Atlanta, GA, USA).

The patients were provided with a new interim crown, which was randomly selected from the following printing angles: 0, 45, and 90 degrees. The patients underwent relining, occlusal adjustment, and polishing of the oral cavity. For relining, self-cured resin (Unifast III; GC Korea, Seoul, Korea) was used. The interim crown surface was scanned before use. The patients had the new interim crown in place for one week. At the next hospital visit, the existing interim crown surface was scanned, and one of the remaining interim crowns was chosen at random and placed. This step was repeated for the placement of the final crown.

The patients, thus, used four crowns in total (conventional method, n = 1; 3D-printing method, n = 3) for one week each. The surfaces of the interim crowns were scanned before and after use. Finally, the technician made a final impression and set the final prosthesis.

**Figure 3:**
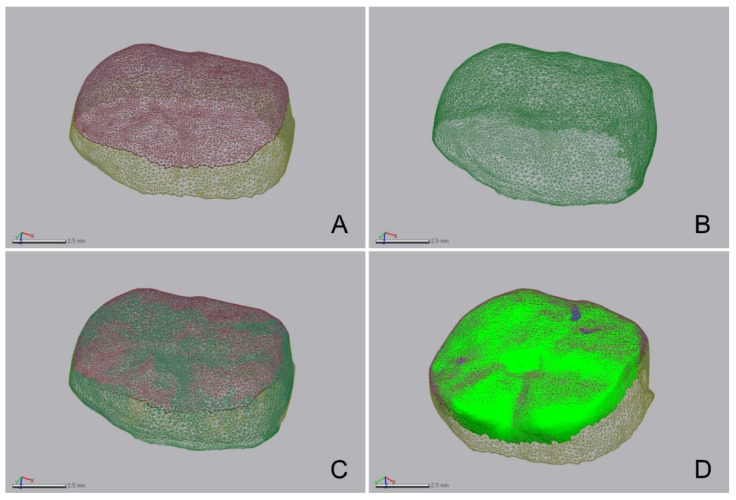
3D analysis procedure. (**A**) Segmented virtual model of the interim crown before wear. (**B**) Virtual model of the interim crown after wear. (**C**) Superimposition. (**D**) Evaluation of wear on the segmented occlusal surface of (**A**).

Using 3D inspection software (Geomagic Control X; 3D Systems, Cary, NC, USA), STL file changes of the interim crowns before and after wear were imported (Figure 3). The volume and height of the interim crowns were measured. The interim crowns before wear were designated as the reference, and the best-fit alignment of those after wear was determined based on the outer surface area of the interim crown, excluding the occlusal surface area before wear (Figure 3). To verify the coincidence of the outer surface area in which the interim crowns overlapped before and after wear, 3D inspection software was used (Geomagic Control X; 3D Systems). The overlapping areas of the two models were found to have a very high coincidence (3.60 ± 0.80 µm). The volume loss was calculated by comparing the volume before and after wear. The root mean square (RMS) was used to calculate the interval between the data points before and after wear (Figure 3) using the following formula:

(1) $$RMS=\frac{1}{\sqrt{n}}\;\cdot \;\sqrt{\sum_{i=1}^{n}{\left(X_{1,i}-X_{2,i}\right)}^{2}}$$

where $X_{1,i}$ is the interim crown’s point cloud before wear, $X_{2,i}$ is the point cloud after wear (which indicates the 3D position of the ith measurement point), and *n* is the number of all point clouds evaluated.

The RMS value showed the difference in the deviation from zero between the two datasets. Thus, a low RMS value indicated a high degree of 3D agreement with the overlapped data. A 3D comparison is shown using a color difference map, and a range of −100 µm and a tolerance range of −10 µm were designated (green).

The statistical analyses in this study were conducted using SPSS Statistics (IBM Co., Armonk, NY, USA). The normal data distribution was first investigated using the Shapiro–Wilk test, and the data were found to be normally distributed. Each group’s wear volume was compared using a one-way analysis of variance test, and an ex-post analysis was conducted using Tukey’s honestly significant difference (HSD) test (α = 0.05).

## 3. Results

**Figure 4:**
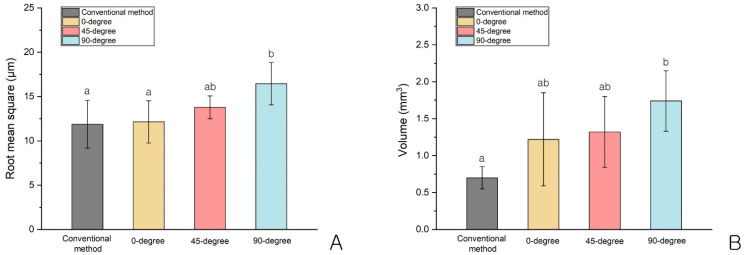
Comparison of the root mean square and wear volume according to build angle: (**A**) root mean square and (**B**) wear volume. The letters (a and b) were determined using Tukey’s HSD test; *p* < 0.05.

Significant differences in the RMS values between the groups were found (*p* = 0.002; Table 3, Figure 4). Compared with the RMS values of the conventional method (11.88 ± 2.69 µm) and the 3D-printing method at 0 degrees (12.14 ± 2.38 µm), the RMS values were significantly higher at 90 degrees (16.46 ± 2.39 µm) (*p* < 0.05; Table 3, Figure 4). Likewise, there was a significant difference in the change in volume between the groups (*p* = 0.002; Table 3, Figure 4), with a significantly higher volume change value at 90 degrees (1.74 ± 0.41 mm^3^) than in the conventional method (0.70 ± 0.15 mm^3^) (*p* < 0.05; Table 3, Figure 4). In addition, the interim crowns fabricated using the conventional method showed significantly lower RMS and volume change values than those fabricated using 3D printing (*p* < 0.05; Table 3 and Table 4, Figure 4).

**Figure 5:**
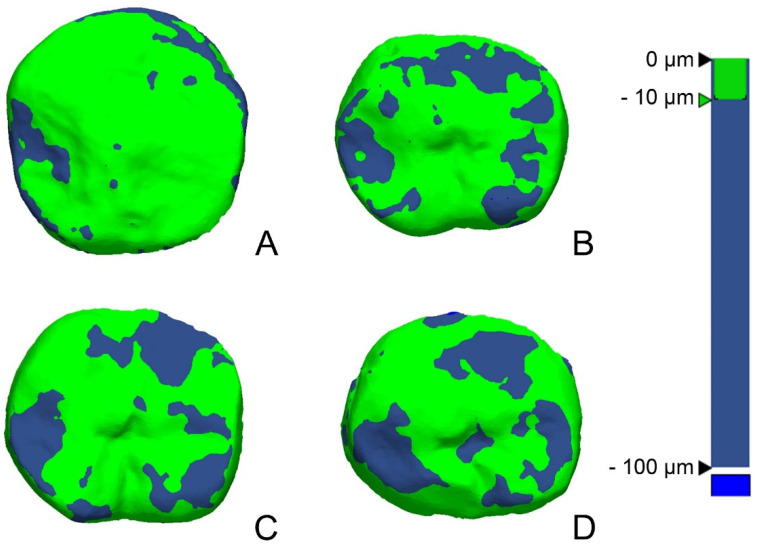
Comparison of 3D wear according to build angle. (**A**) Conventional method. (**B**) 3D-printing build angle of 0 degree. (**C**) 3D-printing build angle of 45 degrees. (**D**) 3D-printing build angle of 90 degrees.

The color difference map according to the build angle showed relatively smaller areas of wear with the conventional method, whereas, with the 3D-printing method, the areas of wear tended to broaden from 0 to 90 degrees (Figure 5). The areas of wear in the color difference map corresponded with the occlusal points found in the oral cavity.

## 4. Discussion

The purpose of this study was to examine wear volume differences according to the printing angle of interim crowns using a DLP 3D-printing method in vivo. We found a significant difference in the RMS values but no significant differences in wear volumes for the 3D-printed interim crowns according to the angle. Additionally, compared with those of the conventional method, there were significantly higher RMS values and wear volumes at 90 degrees (*p* < 0.05). Thus, the null hypothesis that the wear volume of the interim crowns would not be significantly different according to the build angle was rejected.

In this study, a difference in the wear of the interim crowns between the conventional and 3D-printing methods was found (*p* = 0.020). Loss in vertical height can be estimated using the RMS value, while the absolute wear volume can be measured using the volume loss value. The height and wear volume of the interim crowns printed at 0 degrees for the two criteria were the smallest, while the height and wear volume of the interim crowns printed at 90 degrees were the largest. In other words, the greater the printing angle, the greater the wear.

If the volumetric variation is large due to wear, it may cause a loss in vertical dimension and movement of the antagonist in cases of poor periodontal condition. In addition, in cases of anterior restoration, changes in occlusal scheme, including frontal and lateral guidance, may occur, and unnecessary interference may occur. Therefore, the properties of an interim crown require adequate wear resistance.

Differing physical properties according to angle are called anisotropy. A previous study found anisotropy in 3D-printed restorations using a DLP method [20]. However, conflicting results have been reported. One study showed a potentially weak area of polymerization owing to a shadow area between pixels [25]. Additionally, Monzon et al. [25] reported that a resin bar printed at 90 degrees showed superior mechanical properties in terms of flexural modulus and flexural stress compared to those printed at 0 degrees. However, others have suggested that anisotropy is caused by the difference in bonding strength between the inter- and intra-layers [13,26]. Park et al. [13] noted that a three-unit interim bridge printed at 90 degrees had the lowest flexural strength. In addition, in a study of bar-shaped interim material conducted by Kessler et al. [26], the interlayer was found to be the weakest link, and a specimen printed at 90 degrees was more vulnerable to fracture. Thus, our results are similar to those of both Park et al. [13] and Kessler et al. [26], which together suggest that wear decreases as the printing angle decreases and the mechanical properties become stronger. However, additional studies are needed to examine the tendency of wear according to physical properties for more robust conclusions to be drawn.

In the previous study, Kessler et al. [14] reported that the mean wear depth of a DLP-printed interim crown against a metal wheel was between 13 and 66 μm after 50,000 cycles of chewing simulation. Ahn et al. [16] reported that steatite, similar to the surface hardness of enamel, was used as an antagonist, and the wear volume loss of a DLP-printed interim crown was 1.507 mm^3^ after 30,000 cycles of chewing simulation. Hanon et al. [24] reported that the wear depth of non-colored, post-processed DLP cylinder against a steel counterpart was measured between 9 μm and 12 μm among samples of each build angle. Sari. et al. [19] studied an in vivo wear test of interim crowns by milling type and cartridge type on the mandibular first molar for 14 days, and the mean wear data were 296.9 μm (milling type) and 244.4 μm (Cartridge type). However, considering that the thickness of the check film was 21 μm, it could be said that the number of Sari et al.’s experiments was large compared to other articles. As such, the wear data so far have varied depending on the type of opposing tooth, whether they are in vivo or in vitro, the shape of the specimen, and the test period. The results of this experiment were also within the general variation range, and it was possible to show the tendency of the difference in wear in accordance with the build angle. 

There are two types of wear: (1) two-body wear caused by direct friction between the cusps and (2) three-body wear caused indirectly by food between the two cusps. In this study, both types of wear were observed. Two-body wear can be observed through the interim crown’s occlusal points, and the size and number were found to decrease after use in the present study. This can be explained by a decrease in the area where the cusps contacted each other as wear progressed, while the overall occlusal vertical dimension was maintained. However, three-body wear may decrease or increase according to the debris, and the wear volume may be either very small or may not always occur [27]. In fact, after excluding the two-body wear points around the occlusal points, almost no wear could be seen on the color map in the remaining areas (Figure 5), suggesting that the impact of three-body wear was insignificant in this study. In addition, on the color map (Figure 5), the wear areas tended to broaden from 0 to 90 degrees. This again indicated that the greater the printing angle, the greater the wear.

In a study conducted by Beleidy et al. [28], no difference in the wear analysis between 3D scanning and optical digital profilometry (the conventional method) was reported. In the present study, the coincidence of the overlapping outer surface areas of the interim crowns before and after wear was 3.60 ± 0.80 µm, and the two models’ overlapping areas showed very high coincidence.

If the experiment condition in the oral cavity is limited, wear can be evaluated virtually using a finite element method. As a preclinical study, it can reduce the cost and the discomfort of the patient from the clinical trial. For example, Jamari et al. [29] simulated the contact pressure between the metal femoral head and the acetabular cup of an artificial hip joint using a finite element method and proposed a design to reduce cumulative contact pressure and wear in consideration of the physical characteristics of each metal material. Tribst et al. [30] simulated the effect of filling materials and pulp chamber extension on the biomechanical response of an endocrown through a finite element method and proposed an optimal divergence angle with preferred materials to seal the orifice.

To apply this method to interim crown design, it should be preceded by figuring out the physical characteristics of the interim crown, in addition to the build angle and mastication pattern of the jaws. Tian et al. [31] reviewed the factors affecting 3D-printed products as build angle, layer thickness, post-curing, and material composition. Alshamrani et al. [32] reported that a 100 μm layer thickness with a dry storage postprocessing group has the highest flexural strength. Kessler et al. [14] reported that the filler content influenced the wear behavior. As a result, it would be possible to make an optimal crown design for each angle by reflecting the properties of the materials and printing process in a computational simulation. 

In the present study, there was no statistical difference in wear volume between the interim crowns fabricated using the conventional method and those printed at 0 degrees (*p* > 0.05). In general, interim crowns fabricated using a DLP method are printed at 0 degrees. Previous studies have shown weaker wear resistance with the DLP method than with conventional fabrication, milling, and SLA methods [33,34]. Nevertheless, other studies have shown wear resistance to be the same [6,14] or better [15,16] with the DLP method than with conventional fabrication, milling, and SLA methods. Even if the interim crowns are fabricated using the same method, wear might differ according to the composition of the resin material used. Kessler et al. [14] reported significant differences in wear among various 3D-printed materials, and therefore, products with a high filler content were recommended. Kessler et al. [26] also studied an evaluation of the flexural strength of three products and noted that, among the various factors assessed, the product characteristics were the most important. Thus, the results of the present study were limited by the products used. Additional studies are needed to evaluate the wear associated with various products.

This study has several major limitations as an in vivo pilot study. First, the scan data used to evaluate wear were acquired using an intraoral scanner. However, since the volumes of crowns were measured outside the oral cavity rather than intraorally, it was generally more accurate than the degree of suspension presented by an intraoral scanner. In fact, Huang et al. [35] reported 8.66 ± 0.40 µm trueness and 5.44 ± 0.52 µm precision when using the same scanner (CS3600) extraorally. Additionally, Ahn et al. [16] also measured the wear volume of specimens using an intraoral scanner outside the oral cavity. Nevertheless, if a lab scanner was used, it would have been possible to measure the amount of wear more accurately. Second, the wear test was conducted for one week on each interim crown. Previous in vitro studies have been variously tested at levels such as 10,000, 30,000, and 50,000 cycles, as well as up to 200,000 cycles for long term use. In general, 20,000 cycles of a chewing simulator is equivalent to 1 month of use [14,15,36]. Thus, although the wear volume loss for one week would be small, in actual clinical practice, the period of use of an interim crown for a single crown is generally from one to two weeks. In addition, as each participant had to test for each of the four groups in this experiment, the period of use was limited to the shortest period as one week. Third, this experiment was for single-crown restoration. As the overall occlusal vertical dimension was always maintained, it was difficult for the two-body wear to occur continuously by the antagonist, even if the period were further extended. Therefore, the actual measurable change in wear volume was limited. Fourth, anatomical interim crowns were used in this study. While the actual printing angle may have been 45 degrees, for instance, the actual contact angle between the tooth and the temporary restoration could have been greater than or less than 45 degrees considering the cusp shape and direction of jaw movement. In other words, the actual angle may have been altered from the intended angle. Lastly, the masticatory force, dietary habits, and oral habits were individually different. Koc et al. [37] reported that the maximum bite force is higher in males than in females. In addition, although the correlation between age and bite force seems to be significant, it might be assumed that the effect of age on bite force is relatively small. It is practically impossible to avoid individual differences, but to control the variables, each participant in this study was asked to use all four types of interim crown so that the same person’s masticatory force was applied to the interim crown for each angle. In addition, patients who need a treatment on same area were recruited, and the order of use of interim crown was randomly allocated for each angle. 

It is recommended that further studies reflect the following. First, patients who need a long-term restoration or a full-arch restoration with changeable vertical dimensions should be recruited. Second, by increasing the number of samples, participants should be divided into four groups, and interim crowns of one type of angle should be assigned. Thereafter, the wear amount for each group could be compared. Alternatively, patients could be recruited based on the same age and gender. Third, it is necessary to verify the wear resistance by each restoration area. Compared to the posterior teeth, the anterior teeth have a different contact angle on the lingual surface because more complex occlusal contacts, such as lateral guidance, occur. Lastly, since the product content is different for each commercial product, verification is required. 

This study was the first in vivo study on the wear resistance of DLP-printed interim crowns that investigated wear resistance in accordance with the build angle of the DLP-printed interim crowns. The clinical feasibility of DLP-printed interim crowns was verified, and a build angle for wear resistance was suggested. In the future, it could be used to manufacture more complex prostheses, such as anterior restorations and full-arch restorations.

## 5. Conclusions

The interim crowns fabricated using the conventional method showed wear resistance similar (at 0 degree) or superior to those fabricated using the DLP 3D-printing method. The clinical feasibility of DLP-printed interim crowns was verified. To ensure high wear resistance, a printing angle of 90 degrees is not recommended when an interim crown is fabricated using DLP 3D printing; rather, a printing angle of 0 degrees is recommended. Further experiments with longer periods of use, larger numbers of participants, more complex and multi-positioned restorations, the use of precise scanners, and more diverse product groups are needed.

## Figures and Tables

**Table 1:** The profiles of the participants.

No.	Sex	Age	Abutment	Antagonist	Endo.	Etc.
1	F	23	#46	#15, 16	O	-
2	M	31	#36	#25, 26	O	-
3	F	57	#37	#26, 27	O	-
4	F	20	#47	#16, 17	O	-
5	F	41	#36	#25, 26	O	-
6	M	24	#37	#26, 27	O	Dropped out owing to frequent crown fracture.

**Table 2:** Interim crown material.

Group	Product Name	Method	Manufacturer	LOT Number
Conventional group	UNIFAST III	Self-cured	GC Corporation, Tokyo, Japan	2004171
3D-printing group	RAYDENT C&B	DLP 3D printing	Ray Co., Ltd., Hwaseong-si, Korea	RCB209082B

**Table 3:** Comparison of the wear of interim crowns fabricated according to build angle (RMS (µm)).

Build angle	Mean	SD	95% Confidence Interval		Minimum	Maximum	Comparison **
			Lower	Upper			
Conventional method	11.88	2.69	8.53	15.22	7.70	14.80	A
0 degree	12.14	2.38	9.17	15.10	9.7	15.50	A
45 degrees	13.78	1.29	12.17	15.38	11.80	15.10	AB
90 degrees	16.46	2.39	13.49	19.42	13.20	18.90	B
F	4.363						
*p*	0.02 *						

RMS, root mean square; SD, standard deviation. * Significance was determined using one-way ANOVA; *p* < 0.05. ** The letters (A and B) were determined using Tukey’s HSD test; *p* < 0.05.

**Table 4:** Comparison of the wear of interim crowns fabricated according to build angle (volume (mm^3^)).

Build Angle	Mean	SD	95% Confidence Interval		Minimum	Maximum	Comparison **
			Lower	Upper			
Conventional method	0.70	0.15	0.50	0.89	0.50	0.90	A
0 degree	1.22	0.63	0.43	2.00	0.20	1.70	AB
45 degrees	1.32	0.48	0.71	1.92	0.60	1.90	AB
90 degrees	1.74	0.41	1.22	2.25	1.30	2.4	B
F	4.367						
*p*	0.020 *						

RMS, root mean square; SD, standard deviation. * Significance was determined using one-way ANOVA; *p* < 0.05. ** The letters (A and B) were determined using Tukey’s HSD test; *p* < 0.05.

## Data Availability

The datasets used and analyzed during the current study are available from the corresponding author on reasonable request.
